# A claims data-based comparison of comorbidity in individuals with and without dementia

**DOI:** 10.1186/1471-2318-14-10

**Published:** 2014-01-28

**Authors:** Kathrin Bauer, Larissa Schwarzkopf, Elmar Graessel, Rolf Holle

**Affiliations:** 1 Institute of Health Economics and Health Care Management, Helmholtz Zentrum München, Ingolstaedter Landstrasse 1, 85764 Neuherberg, Germany; 2 Department of Medical Psychology and Medical Sociology, Alexander-Universität Erlangen-Nürnberg, Clinic for Psychiatry and Psychotherapy, Schwabachanlage 6, Erlangen 91054, Germany

**Keywords:** Elderly, Multimorbidity, Disease groups, Gender, Care setting, Administrative data

## Abstract

**Background:**

Multimorbidity is common in advanced age, and is usually associated with negative – yet to some extent preventable – health outcomes. Detecting comorbid conditions is especially difficult in individuals with dementia, as they might not always be able to sufficiently express discomfort. This study compares relevant comorbidity complexes in elderly people with and without dementia, with a particular look at gender- and living environment-specific differences. Moreover, associations between selected comorbid conditions and dementia are reviewed more closely.

**Methods:**

Using 2006 claims data from a large German Statutory Health Insurance fund, 9,139 individuals with dementia and 28,614 age- and gender-matched control subjects aged 65 years and older were identified. A total of 30 comorbidity complexes were defined based on ICD-10 codes. Corresponding prevalence rates were calculated, and the association between a distinct condition and dementia was evaluated via logistic regression in the overall sample as well as in analyses stratified by gender and living environment.

**Results:**

Individuals with dementia were more likely to be diagnosed with 15 comorbidity complexes, including Parkinson’s, stroke, diabetes, atherosclerosis (supposed dementia risk factors) or fluids and electrolyte disorders, insomnia, incontinence, pneumonia, fractures and injuries (supposed sequelae). In contrast, they were less likely to be diagnosed with 11 other conditions, which included vision and hearing problems, diseases of the musculoskeletal system, lipoprotein disorders and hypertension. In a gender-stratified analysis, the patterns remained largely the same, but a bigger comorbidity gap between cases and control subjects emerged in the male population. Restricting the analysis to community-living individuals did not lead to any substantial changes.

**Conclusion:**

Besides strengthening the evidence on accepted dementia risk factors and sequelae, the analyses point to particular conditions that are likely to remain untreated or even undiagnosed. This issue seems to affect male and female individuals with dementia to varying degrees. Raising awareness of these conditions is important to possibly preventing comorbidity-associated complications and disease progression in dementia patients. To more comprehensively understand the mutual interactions between dementia and comorbidity, further research on diagnostic and treatment attitudes regarding comorbidity in dementia patients and on their gender-specific health-seeking behaviour seems to be required.

## Background

With a growing number of elderly people in Germany, the occurrence of age-related diseases is increasing steadily. Dementia is considered to be one of the most challenging of these, not only for those affected, but also for their relatives and formal caregivers. The term dementia refers to a collection of symptoms with different underlying causes that are not yet completely understood. Presumably, pathological changes and lesions involving various brain areas and neuronal networks lead to changes in the functioning of the brain, which in consequence affect memory, activities of daily living (ADL) and the behaviour of patients [1,2].

Multimorbidity is also a well-known phenomenon in the elderly. According to a recent German study, about 62% of the population aged 65 years and older is multimorbid [3]. Yet, findings about whether the amount and type of comorbidity differ between individuals with and without dementia are inconclusive. Whereas an older study suggests that Alzheimer patients are healthier [4], some authors find no differences in comorbidity burden [5,6]. Other studies find a higher comorbidity burden in dementia patients [7,8] or report mixed findings, suggesting that some diseases are more and others less frequent in individuals with dementia compared with non-demented control subjects [9,10].

Multimorbidity is associated with a greater risk of dying, poor functional status, reduced quality of life and greater use of health care services [11]. It is therefore important to recognize and manage comorbid conditions well, so that they do not worsen a person’s health status. Dementia patients, in particular in the advanced stages, may have difficulty in communicating their symptoms. In addition, if dealing with a severe health problem such as dementia, physicians might lose sight of other conditions. In consequence, dementia patients could be at risk of being underdiagnosed for comorbid diseases. This hypothesis is confirmed by Löppönnen et al., who found more undiagnosed diseases in patients with dementia than in control subjects [12].

Highlighting relevant comorbidity in dementia patients will help to alert physicians and, consequently, to improve the prevention and treatment of complications in a patient group that might not always be able sufficiently to express discomfort. It is also important to take a closer look at the reasons for observed comorbidity differences. Different prevalence rates of diagnosed comorbidity might be explained to some extent by a (dementia-related) change in pathophysiological mechanisms, which eventually results in different risks of individuals with and without dementia for developing a distinct comorbid condition. In this case, different prevalence rates of documented comorbidity reflect a really existing – and medically reasonable – difference regarding the occurrence of a comorbid condition. However, differences in documented prevalence might also be explained to some extent by different health care-seeking behaviour or a different attitude towards health care service provision for individuals with and without dementia, and hence more likely reflect under-diagnosis and under-treatment of conditions with a comparable real prevalence rate.

Cross-sectional population-based studies can be considered as some kind of gold standard to assess the prevalence of comorbidity in individuals with and without dementia as they include a representative sample of individuals at risk. However, this comprehensive research approach is rarely chosen [12]. Instead, most previous studies drew conclusions on comorbidity in individuals with dementia based on hospital admission and discharge data [4,5,7,9,10], which does not reflect true prevalence rates, owing to the fact that only a small proportion of all patients undergoes hospital treatment. To this end, insurance claims data, which include all individuals who seek any kind of inpatient or outpatient treatment [6,8], seem to be a less selective approach, as less severe treatment episodes (which do not require hospitalization) are also accounted for.

Therefore, we decided to use claims data from a large regional German Statutory Health Insurance (SHI) fund to: (1) investigate the prevalence of various diagnosed comorbidity complexes in dementia patients compared with control subjects; (2) explore whether the comorbidity pattern changes in a gender-specific analysis; and (3) identify differences according to living environment.

## Methods

### Data source and sample selection

Analyses are based on claims data from AOK Bavaria, a large regional German SHI fund, which covers about 50% of the resident population aged 65 years and older in the study region. Complete data from 2005 to 2007 were provided for all insurants born before 1941 and continuously insured until 2007.

Dementia cases were identified from 2005 and 2006 data via in- and outpatient diagnoses and anti-dementia drug prescriptions. As in the German SHI system physician diagnoses always refer to a distinct quarter of the year, each quarter of the years 2005 and 2006 was screened for the documentation of dementia-specific diagnoses (ICD-10 codes ‘F00’, ‘F01’, ‘F02’, ‘F03’ and ‘G30’) or anti-dementia drugs ((ATC codes N06DA (cholinesterase inhibitors) and N06DX01 (memantine)). To improve the validity of diagnoses, the analyses accounted only for those individuals who had at least one corresponding dementia indicator during three out of four consecutive quarters. Non-demented control subjects were randomly selected from the remaining insurants without any dementia indicator in 2005 and 2006. They were matched by age based on year of birth and gender in a 4:1 ratio, with a slightly inexact matching beyond the age of 82 years on account of the high dementia prevalence in these age groups. Following this approach, 9,147 dementia patients and 29,741 control subjects were identified. A detailed description of the selection process can be found elsewhere [13].

Our analyses rely on 2006 data. As prevalent comorbidity is the focus of our analyses, we only included individuals with at least one documented ICD-10 comorbidity code in 2006, and 9,139 dementia cases (99.9%) and 28,614 control subjects (96.2%) remained.

For the living environment-specific analysis, the institutional and the community setting were distinguished based on payments for a component of long-term care insurance (LTCI) called ‘institutional care’, which is the only parameter within German claims data to indicate an individual’s care setting. This service domain is only available for individuals who are institutionalized within nursing homes or the special wards of residential homes for the elderly. Assuming that institutional care is only affordable with LTCI coverage, individuals receiving no LTCI support at all or no LTCI support for ‘institutional care’ fall into the community setting. Subjects with an uninterrupted sequence of LTCI support for ‘institutional care’ in 2006 were allocated to the institutional setting, and subjects for whom the starting date of payments for institutional care was in 2006 were classified as transferring to a nursing home. Details about the allocation to settings are available elsewhere [14]. After excluding institutionalized individuals as well as transferring individuals, 5,524 cases and 27,595 control subjects were suitable for the community-setting analysis. However, our approach does not allocate residents of homes for the handicapped to the institutional setting.

Need for care within the different settings is described by ‘care levels’, which indicate nursing dependency. Three levels are distinguished that define how often and for how long assistance with ADL is needed [15]. People with the highest nursing dependency are assigned to level 3, and need for care decreases with each level.

### Diagnosis groups

We analysed the prevalence of comorbid conditions based on 2006 inpatient and outpatient diagnoses, which were grouped into 30 comorbidity complexes.

The choice of comorbidity complexes is based on a list of 46 chronic conditions compiled by Schäfer et al. [16], who provide a broad range of chronic disease groups. Because we also wanted to cover the non-chronic but acute presence of these conditions (e.g. N17: acute renal failure), Schäfer’s groups were amended with the respective ICD-10 codes. Additionally, single ICD-10 codes disregarded by Schäfer et al., but highly prevalent (prevalence >10%) in our sample, were assessed. Some of these were added to existing complexes, and some formed new complexes and were added to the list of conditions, as well as some groups that were added after a review of disease groups commonly chosen by other authors [5-10,12,16-23]. As a result, 52 comorbidity complexes were assembled and reviewed.

For our analyses, we included all complexes that had (1) a prevalence of at least 15% within the case or control group of our study population or were (2) accounted for within at least five of the reference studies [5-10,12,16-23] or were (3) considered as being of significant medical interest within a thorough appraisal by the authors. Multiple mentioning of conditions was allowed (e.g. highly prevalent, and frequently mentioned in previous studies). Using this approach, 30 comorbidity complexes were finally considered. The source of origin of these 30 comorbidity complexes is accessible as Additional file 1.

For the main analysis, a person was allocated to a diagnosis group if a corresponding diagnosis was documented at least once in 2006 in order to capture acute conditions as well. Additionally, sensitivity analyses with more restrictive diagnosis documentation requirements (coding of a diagnosis in at least two or four quarters of 2006) were run.

The overall burden of comorbidity was measured by the Charlson Index (CI) using the ICD-10 comorbidity coding algorithm from Quan et al. [24]. The higher the index, the higher the comorbidity burden. The original version of the CI includes 17 disease categories with dementia being one of them. Thus, dementia patients with an equal comorbidity profile to non-demented control subjects would have a higher CI because dementia contributes to calculating the index score. To reflect disease burden apart from dementia, we calculated a modified version of the CI, which consists of only 16 disease categories and does not include dementia in calculating the index score.

### Statistical analysis

Descriptive statistics characterize the study population. The proportion of affected dementia patients and control subjects was calculated for each comorbidity complex. To investigate the association between a comorbidity complex and dementia, the likelihood of being affected by a comorbidity complex in the presence of dementia in comparison with a control subject was calculated using binary logistic regression. Because of the mentioned inexact matching and distortion as a result of restriction of the sample to people with at least one documented ICD-10 code in 2006, the analysis was adjusted for age and gender.

Odds ratios (ORs), 95% confidence intervals (CI) and p-values are provided. As we tested 30 comorbidity complexes, we addressed multiple testing by setting a Bonferroni-adjusted significance level of p < 0.0017 (0.05/30).

To evaluate the impact of gender, a dementia–gender interaction term was added to the model and, as the interaction terms were significant for the majority of comorbidity complexes, we also chose to display the results of a gender-stratified analysis, which is adjusted for age only.

Statistical Analysis System (SAS) 9.2 was used for data analysis.

## Results

### Baseline characteristics

The study population included 37,753 individuals aged between 65 and 103 years. Some 27,012 (71.6%) individuals were female, and 33,119 individuals (87.7%) lived within the community setting during the entire observation period.

Table 1 displays the demographic characteristics of the study population as a whole as well as according to dementia status. Comparing individuals with and without dementia revealed that the share of permanently community-living individuals was significantly higher in non-demented control subjects (96.4% vs. 67.9%). Corresponding to this, 88.3% of control subjects showed no impairment in ADL (no care level), whereas only 37.9% of dementia patients hardly needed assistance at all (p <0.0001). The modified CI was significantly higher in the case group (p <0.0001) and significantly higher for men than for women in both groups (p <0.0001 each).

**Table 1:** Baseline characteristics of 2006 study sample

	**Entire sample**	**Case group**	**Control group**	p-value***
**Number:** N	37,753	9,139	28,614	
**Age:** mean (SD)	80.1 (6.8)	81.6 (7.4)	79.6 (6.4)	<0.0001
**Gender:** females (%)	27,012 (71.6)	6,814 (74.6)	20,198 (70.6)	
**Environment:** n (%)				<0.0001
Living in community setting	33,119 (87.7)	5,524 (60.4)	27,595 (96.4)	<0.0001
Living in nursing home	3,646 (9.7)	2,934 (32.1)	712 (2.5)	
Change in environment	988 (2.6)	681 (10.3)	307 (1.1)	
Care level*: n (%)				<0.0001
None	28,746 (76.1)	3,466 (37.9)	25,280 (88.3)	
1	42,066 (11.1)	2,066 (22.6)	2,140 (7.5)	
2	3,131 (8.3)	2,164 (23.7)	967 (3.4)	
3	1,670 (4.4)	1,443 (15.8)	227 (0.8)	
Modified Charlson Index**: mean (SD)	3.2 (2.7)	3.6 (2.7)	3.0 (2.7)	<0.0001

SD = Standard Deviation. *Care level corresponds to the care level on 30 June 2006.

**The Charlson Index (CI) category ‘dementia’ is set at zero for all individuals.

***P-values derived from Chi^2^ test for distinct variables and from Kruskal–Wallis test for continuous variables.

### Range of comorbidity

Table 2 displays the comorbidity complexes. In both, the case and the control group, hypertension was the most common comorbidity complex: it was documented for 73.4% of dementia patients and 77.8% of control subjects. Hypertension was followed by lower back pain in the control group (58.9%) and incontinence (50.3%) in the case group. The prevalence of disorders directly related to dementia development (Parkinson’s, stroke) was increased for dementia patients, as well as the prevalence of diseases that are related to loss of cognitive abilities (incontinence, electrolyte and fluid imbalances). Also, fractures and injuries, pneumonia, mental and behavioural disorders, anaemia, diabetes as well as cardiac and renal insufficiency were observed more often in dementia patients. In contrast, problems with the musculoskeletal system, vision and hearing were documented less frequently for cases. Cancer, thyroid dysfunction, lipoprotein disorders, hypertension, arrhythmias and varicosis were also less frequent in cases than in control subjects. This is reflected in the results of the logistic regression, with high ORs for Parkinson’s disease (5.21), incontinence (3.01), cerebral ischaemia/chronic stroke (2.59), pneumonia (2.31) and depression (2.08) and low ORs for vision reduction (0.59), lower back pain (0.69), lipoprotein disorders (0.72), joint arthrosis (0.74) and hypertension (0.76). The sensitivity analyses did not show substantial changes in the results for most of the groups; in particular, there were no substantial changes in ORs.

**Table 2:** Diagnosed comorbidity complexes of dementia patients and control subjects

**Diagnosis group**	**Case n (%) N = 9,139**	**Control n (%) N = 28,614**	OR* (95% CI)	p-value**
**Neoplasms**				
Cancer (all) (C00–D48)	2,284 (25.0)	7,880 (27.5)	0.89 (0.85–0.94)	**<0.0001**
**Diseases of the blood and blood-forming organs**				
Anaemia (D50–53, D55–64)	2,321 (25.4)	5,518 (19.3)	1.40 (1.32–1.48)	**<0.0001**
**Endocrine, nutritional and metabolic diseases**				
Thyroid dysfunction (E01–07)	2,266 (24.8)	8,027 (28.1)	0.86 (0.81–0.91)	**<0.0001**
Diabetes (E10–14)	4,135 (45.2)	11,252 (39.3)	1.29 (1.23–1.35)	**<0.0001**
Disorders of lipoprotein metabolism and other lipidaemias (E78)	3,797 (41.5)	14,685 (51.3)	0.72 (0.69–0.76)	**<0.0001**
Fluids/electrolyte disorders (E86, E87, R60)	3,175 (34.7)	5,712 (20.0)	2.01 (1.91–2.12)	**<0.0001**
**Mental and behavioural disorders**				
Psychotic/neurotic disorders (F20–29, F40–48)	2,189 (24.0)	5,122 (17.9)	1.53 (1.45–1.62)	**<0.0001**
Depression (F32–33)	2,790 (30.5)	5,017 (17.5)	2.08 (1.97–2.20)	**<0.0001**
Insomnia (F51, G47)	1,485 (16.2)	3,504 (12.2)	1.34 (1.26–1.43)	**<0.0001**
**Diseases of the nervous system**				
Parkinson’s disease (G20–22)	1,203 (13.2)	805 (2.8)	5.21 (4.75–5.72)	**<0.0001**
**Diseases of the eye and ear**				
Severe vision reduction (H17–18, H25–28, H31, H33, H34.1–34.2, H34.8–34.9, H35–36, H40, H43, H47, H54)	3,406 (37.3)	14,254 (49.8)	0.59 (0.57–0.62)	**<0.0001**
Severe hearing loss (H90, H91)	1,650 (18.1)	5,267 (18.4)	0.91 (0.86–0.97)	0.004
**Diseases of the circulatory system**				
Hypertension (I10–15)	6,704 (73.4)	22,266 (77.8)	0.76 (0.72–0.80)	**<0.0001**
Coronary artery disease (CAD) (I20–25)	3,638 (39.8)	11,018 (38.5)	1.01 (0.96–1.06)	0.821
Cardiac arrhythmias (I44–49)	2,570 (28.1)	8,229 (28.8)	0.92 (0.87–0.97)	**0.002**
Cardiac insufficiency (I50)	4,233 (46.3)	9,277 (32.4)	1.58 (1.50–1.66)	**<0.0001**
Atherosclerosis/peripheral arterial occlusive disease (I65–66, I67.2, I70, I73.9)	3,945 (43.2)	8,838 (30.9)	1.64 (1.56–1.72)	**<0.0001**
Cerebral ischaemia/chronic stroke (G45, I60–64, I69)	2,960 (32.4)	4,352 (15.2)	2.59 (2.45–2.74)	**<0.0001**
Lower limb varicosis (I83, I87.2)	2,093 (22.9)	7,316 (25.6)	0.85 (0.80–0.89)	**<0.0001**
**Diseases of the respiratory system**				
Pneumonia (J12–18)	1,062 (11.6)	1,497 (5.2)	2.31 (2.12–2.51)	**<0.0001**
Asthma/chronic obstructive pulmonary disease (COPD) (J40–47)	2,403 (26.3)	7,273 (25.4)	1.05 (1.00–1.11)	0.060
**Diseases of the musculoskeletal system and connective tissue**				
Arthritis (M02, M05–06, M08, M10, M11–13)	1,035 (11.3)	3,932 (13.7)	0.82 (0.76–0.88)	**<0.0001**
Joint arthrosis (M15–19)	3,746 (41.0)	13,524 (47.3)	0.74 (0.71–0.78)	**<0.0001**
Purine/pyrimidine metabolism disorders/gout (E79, M10)	2,291 (25.1)	8,141 (28.5)	0.86 (0.82–0.91)	**<0.0001**
Lower back pain (M40–48, M50–54)	4,474 (49.0)	16,857 (58.9)	0.69 (0.66–0.72)	**<0.0001**
Osteoporosis (M80–82)	2,178 (23.8)	6,465 (22.6)	1.00 (0.95–1.06)	0.937
**Diseases of the genitourinary system**				
Renal insufficiency/failure (N17–19)	1,978 (21.6)	4,667 (16.3)	1.36 (1.28–1.44)	**<0.0001**
Incontinence (N39.3, R32, R15)	4,594 (50.3)	6,914 (24.2)	3.01 (2.87–3.17)	**<0.0001**
**Fractures, injuries and fall risks**				
Fractures and injuries (S00–T14)	3,965 (43.4)	8,958 (31.3)	1.60 (1.52–1.68)	**<0.0001**
Fall risk and dizziness (R26, R29.6, R42, H81, H82)	2,251 (24.6)	5,761 (20.1)	1.23 (1.16–1.30)	**<0.0001**

*ORs are adjusted for age and sex.

**Bold p-values considered significant according to Bonferroni adjustment.

### Gender-stratified case–control comparison

As pointed out in Table 3, females with and without dementia were older than their male counterparts (p <0.0001 for both) and, in both groups, fewer women than men stayed in the community setting (p <0.0001 for both).

**Table 3:** Gender-specific baseline characteristics of 2006 study sample

		**Male**				**Female**		
**Characteristics**	**All**	**Case**	**Control**	p-value***	**All**	**Case**	**Control**	p-value***
**Number:** N	10,741	2,325	8,416		27,012	6,814	20,198	
**Age:** mean (SD)	77.7 (6.7)	78.2 (7.2)	77.5 (6.6)	<0.0001	81.0 (6.5)	82.8 (7.2)	80.4 (6.2)	0.0009
**Environment:** n (%)				<0.0001				<0.0001
Living in community setting	9,973 (92.8)	1,684 (72.4)	8,289 (98.5)		23,146 (85.7)	3,840 (56.4)	19,306 (95.6)	
Living in nursing home	562 (5.2)	482 (20.7)	80 (1.0)		3,084 (11.4)	2,452 (36.0)	632 (3.1)	
Change in environment	206 (1.9)	159 (6.8)	47 (0.6)		782 (2.9)	522 (7.7)	260 (1.3)	
Care level*: n (%)				<0.0001				<0.0001
None	8,823 (82.1)	1,047 (45.0)	7,776 (92.4)		19,923 (73.8)	2,419 (35.5)	17,504 (86.7)	
1	926 (8.6)	518 (22.3)	408 (4.8)		3,280 (12.1)	1,548 (22.7)	1,732 (8.6)	
2	708 (6.6)	512 (22.0)	196 (2.3)		2,423 (9.0)	1,652 (24.2)	771 (3.8)	
3	284 (2.6)	248 (10.7)	36 (0.4)		1,386 (5.1)	1,195 (17.5)	191 (0.9)	
CI**: mean (SD)	3.7 (3.0)	4.3 (3.0)	3.5 (3.0)	<0.0001	3.0 (2.5)	3.4 (2.5)	2.8 (2.5)	<0.0001

SD = Standard Deviation. *Care level corresponds to the care level on 30 June 2006.

**The Charlson Index (CI) category ‘dementia’ is set at zero for all individuals.

***P-values derived from Chi^2^ test for distinct variables and from Kruskal–Wallis test for continuous variables.

Table 4 displays gender-specific comorbidity. Considering the Bonferroni adjustment, the dementia–gender interaction terms added to the first model were significant for 14 of the 30 comorbidity complexes (thyroid dysfunction, depression, insomnia, Parkinson’s disease, vision reduction, hypertension, cardiac insufficiency, atherosclerosis/peripheral arterial occlusive diseases, cerebral ischaemia/stroke, lower limb varicosis, joint arthrosis, osteoporosis, incontinence as well as fall risks/dizziness). The tendency of the ORs remained largely constant in both male and female cohorts. When comparing the likelihood of a male dementia patient being diagnosed with a certain comorbidity complex (compared with a control subject) with the likelihood of a female dementia patient for the same complex, the OR of the male cohort almost always exceeded the corresponding OR in the female cohort. For instance, large differences were observed for Parkinson’s (6.64 male vs. 4.71 female), incontinence (4.19 male vs. 2.71 female), stroke (3.32 male vs. 2.34 female), depression (2.81 male vs. 1.93 female) and vision reduction (0.73 male vs. 0.56 female).

**Table 4:** Diagnosed comorbidity complexes of dementia patients and control subjects stratified by gender

	**Men (N = 10,741; 2,325 cases; 8,416 control subjects)**			**Women (N = 27,012; 6,814 cases; 20,198 control subjects)**			
**Diagnosis group**	**Case (%)**	**Control (%)**	**OR**	**Case (%)**	**Control (%)**	OR*	p interaction**
**Neoplasms**							
Cancer (all)	33.9	35.9	0.90 (0.82–0.99)	22.0	24.1	0.91 (0.85–0.97)	0.589
**Diseases of the blood and blood-forming organs**							
Anaemia	26.1	20.0	1.40 (1.26–1.56)	25.2	19.0	1.40 (1.31–1.50)	0.925
**Endocrine, nutritional and metabolic diseases**							
Thyroid dysfunction	18.0	17.7	1.02 (0.91–1.16)	27.1	32.4	0.83 (0.78–0.88)	**<0.0001**
Diabetes	47.9	40.2	1.38 (1.26–1.51)	44.3	39.0	1.25 (1.18–1.32)	0.109
Disorders of lipoprotein metabolism and other lipidaemias	45.1	51.0	0.81 (0.74–0.89)	40.3	51.5	0.70 (0.66–0.74)	0.008
Fluids/electrolyte disorders	32.2	17.2	2.24 (2.02–2.49)	35.6	21.1	1.94 (1.83–2.06)	0.016
**Mental and behavioural disorders**							
Psychotic/neurotic disorders	20.5	13.5	1.67 (1.48–1.88)	25.1	19.7	1.51 (1.42–1.62)	0.054
Depression	24.5	10.4	2.81 (2.50–3.17)	32.6	20.5	1.93 (1.81–2.05)	**<0.0001**
Insomnia	14.9	9.6	1.65 (1.44–1.88)	16.7	13.3	1.25 (1.16–1.35)	**<0.0001**
**Diseases of the nervous system**							
Parkinson’s disease	17.0	2.9	6.64 (5.62–7.84)	11.9	2.8	4.71 (4.21–5.28)	**<0.0001**
**Diseases of the eye and ear**							
Severe vision reduction	39.8	47.2	0.73 (0.67–0.80)	36.4	50.9	0.56 (0.53–0.59)	**<0.0001**
Severe hearing loss	20.9	20.8	0.97 (0.87–1.09)	17.1	17.4	0.90 (0.83–0.97)	0.172
**Diseases of the circulatory system**							
Hypertension	71.6	73.2	0.92 (0.83–1.02)	74.0	79.8	0.70 (0.66–0.75)	**<0.0001**
Coronary artery disease (CAD)	45.6	42.9	1.10 (1.00–1.20)	37.8	36.7	0.97 (0.91–1.02)	0.044
Cardiac arrhythmias	33.9	31.6	1.03 (0.94–1.14)	26.5	27.6	0.88 (0.83–0.94)	0.003
Cardiac insufficiency	40.9	26.2	1.90 (1.73–2.10)	48.2	35.0	1.48 (1.39–1.56)	**<0.0001**
Atherosclerosis/peripheral arterial occlusive disease	47.7	32.6	1.86 (1.70–2.05)	41.6	30.2	1.56 (1.48–1.66)	**0.002**
Cerebral ischaemia/chronic stroke	39.1	16.1	3.32 (3.00–3.67)	30.1	15.9	2.34 (2.19–2.50)	**<0.0001**
Lower limb varicosis	17.2	16.6	1.04 (0.92-1.17)	24.8	29.3	0.81 (0.76-0.86)	**<0.0001**
**Diseases of the respiratory system**							
Pneumonia	14.6	6.4	2.45 (2.12–2.83)	10.6	4.7	2.23 (2.02–2.48)	0.353
Asthma/chronic obstructive pulmonary disease (COPD)	34.2	31.0	1.15 (1.04–1.27)	23.6	23.1	1.02 (0.95–1.09)	0.030
**Diseases of the musculoskeletal system and connective tissue**							
Arthritis	13.4	15.3	0.86 (0.76–0.99)	10.6	13.1	0.79 (0.73–0.87)	0.348
Joint arthrosis	36.0	39.6	0.85 (0.77–0.93)	42.7	50.5	0.71 (0.68–0.76)	**0.001**
Purine/pyrimidine metabolism disorders/gout	32.9	36.8	0.85 (0.77–0.93)	22.4	25.0	0.86 (0.81–0.92)	0.581
Lower back pain	48.5	55.7	0.76 (0.69–0.83)	49.1	60.3	0.67 (0.63–0.71)	0.016
Osteoporosis	8.6	6.5	1.33 (1.12–1.57)	29.0	29.3	0.97 (0.91–1.03)	**<0.0001**
**Diseases of the genitourinary system**							
Renal insufficiency/failure	28.3	20.5	1.50 (1.35–1.66)	19.4	14.6	1.30 (1.21–1.40)	0.024
Incontinence	48.3	18.2	4.19 (3.79–4.63)	50.9	26.7	2.71 (2.56–2.87)	**<0.0001**
**Injuries, fractures and fall risks**							
Fractures and injuries	37.3	27.8	1.52 (1.38–1.68)	45.5	32.8	1.63 (1.54–1.73)	0.253
Fall risk and dizziness	22.8	15.4	1.59 (1.42–1.78)	25.2	22.1	1.14 (1.07–1.22)	**<0.0001**

*ORs are adjusted for age.

**P-values for case*gender interaction, when interaction term was added to the model in Table 2; bold p-values considered significant according to Bonferroni adjustment.

### Community setting

After restricting the main analysis to people living in a community setting (Table 5), prevalence rates in the control group hardly changed, as 96.4% of the control population were living in the community setting anyway. For the dementia population, the largest prevalence decrease was observed for incontinence and fluids/electrolyte disorders and the largest increase for back pain and lipoprotein disorders. In the community setting, the likelihood of a dementia patient being diagnosed with a comorbidity complex was higher than in the main analysis, except for Parkinson’s, pneumonia, incontinence, fluids/electrolyte disorders, fractures and injuries, stroke and cardiac insufficiency.

**Table 5:** Diagnosed comorbidity complexes of community-living dementia patients and control subjects

**Diagnosis group**	**n (%) case N = 5,524**	**n (%) control N = 27,595**	OR* (CI)	p-value**
**Neoplasms**				
Cancer (all)	1,514 (27.4)	7,637 (27.7)	0.98 (0.92–1.05)	0.600
**Diseases of the blood and blood-forming organs**				
Anaemia	1,383 (25.0)	5,271 (19.1)	1.40 (1.31–1.50)	**<0.0001**
**Endocrine, nutritional and metabolic diseases**				
Thyroid dysfunction	1,492 (27.0)	7,766 (28.1)	0.96 (0.90–1.03)	0.251
Diabetes	2,534 (45.9)	10,766 (39.0)	1.33 (1.25–1.41)	**<0.0001**
Disorders of lipoprotein metabolism and other lipidaemias	2,667 (48.3)	14,320 (51.9)	0.89 (0.84–0.94)	**<0.0001**
Fluids/electrolyte disorders	1,615 (29.3)	5,315 (19.3)	1.70 (1.60–1.82)	**<0.0001**
**Mental and behavioural disorders**				
Psychotic/neurotic disorders	1,403 (25.4)	4,933 (17.9)	1.62 (1.51–1.73)	**<0.0001**
Depression	1,736 (31.4)	4,708 (17.1)	2.28 (2.14–2.44)	**<0.0001**
Insomnia	928 (16.8)	3,302 (12.0)	1.47 (1.36–1.60)	**<0.0001**
**Diseases of the nervous system**				
Parkinson’s disease	558 (10.1)	704 (2.6)	4.25 (3.78–4.77)	**<0.0001**
**Diseases of the eye and ear**				
Severe vision reduction	2,318 (42.0)	13,826 (50.1)	0.72 (0.68–0.76)	**<0.0001**
Severe hearing loss	1,096 (29.8)	5,057 (18.3)	1.07 (0.99–1.15)	0.076
**Diseases of the circulatory system**				
Hypertension	4,197 (76.0)	21,455 (77.7)	0.90 (0.84–0.96)	**0.002**
Coronary artery disease (CAD)	2,285 (41.4)	10,584 (38.4)	1.11 (1.04–1.17)	**<0.0001**
Cardiac arrhythmias	1,685 (30.5)	7,908 (28.7)	1.06 (1.00–1.13)	0.061
Cardiac insufficiency	2,350 (42.5	8,692 (31.5)	1.56 (1.47–1.66)	**<0.0001**
Atherosclerosis/peripheral arterial occlusive disease	2,494 (45.1)	8,425 (30.5)	1.85 (1.74–1.96)	**<0.0001**
Cerebral ischaemia/chronic stroke	1,651 (29.9)	3,963 (14.4)	2.51 (2.34–2.68)	**<0.0001**
Lower limb varicosis	1,462 (26.5)	7,056 (25.6)	1.05 (0.98–1.12)	0.143
**Diseases of the respiratory system**				
Pneumonia	482 (8.7)	1,375 (5.0)	1.80 (1.61–2.00)	**<0.0001**
Asthma/chronic obstructive pulmonary disease (COPD)	1,472 (26.6)	6,989 (25.3)	1.07 (1.00–1.14)	0.056
**Diseases of the musculoskeletal system and connective tissue**				
Arthritis	712 (12.9)	3,793 (13.7)	0.93 (0.85–1.01)	0.102
Joint arthrosis	2,446 (44.3)	13,046 (47.3)	0.88 (0.83–0.93)	**<0.0001**
Purine/pyrimidine metabolism disorders/gout	1,543 (27.9)	7,872 (28.5)	0.97 (0.91–1.03)	0.322
Lower back pain	3,124 (56.6)	16,377 (59.3)	0.90 (0.85–0.96)	**<0.0001**
Osteoporosis	1,365 (24.7)	6,192 (22.4)	1.14 (1.06–1.22)	**<0.0001**
**Diseases of the genitourinary system**				
Renal insufficiency/failure	1,166 (21.1)	4,409 (16.0)	1.37 (1.27–1.48)	**<0.0001**
Incontinence	2,456 (44.5)	6,392 (23.2)	2.64 (2.49–2.81)	**<0.0001**
**Injuries, fractures and fall risks**				
Fractures and injuries	2,122 (38.4)	8,505 (30.8)	1.38 (1.30–1.47)	**<0.0001**
Fall risk and dizziness	1,524 (27.6)	5,519 (20.0)	1.50 (1.40–1.60)	**<0.0001**

*ORs are adjusted for age and sex.

**Bold p-values considered significant according to Bonferroni adjustment.

A gender-stratified analysis of the community-living population revealed a particularly strong decrease in ORs regarding the first five of these comorbidity complexes for both genders and a particularly strong increase in ORs regarding lower back pain, dyslipidaemia, fall risks/dizziness and lower limb varicosis. Altogether, the percentage change in ORs was in general more pronounced in women than in men. Further information on this gender-stratified analysis is available as Additional file 2.

## Discussion

Based on insurance claims data, we examined the documented prevalence of comorbid conditions in people with dementia and a control population. In the overall sample, people with dementia had a significantly higher chance than control subjects of being diagnosed with a certain comorbid condition for 15 out of 30 investigated comorbidity complexes and a significantly lower chance for 11 of them. In comparison with the control subjects, men with dementia seemed to be affected more frequently than women with dementia for distinct conditions. Restricting the analysis to people living in the community setting did not lead to crucial changes in the results.

To comprehensively judge our findings, different explanatory approaches regarding the observed prevalence rates of documented comorbidity have to be considered. First, the observed associations could be based on common pathology. There are conditions, in particular Parkinson’s disease [25], stroke [2,26,27], vascular risk factors such as diabetes, atherosclerosis and hypertension [28-32] and possibly depression [33,34], that are established risk factors for dementia. Except for hypertension, the chance of being affected by these comorbidity complexes was higher for dementia patients in our study as well. Our finding of an inverse association between hypertension and dementia might be explained by the importance of age at hypertension exposure: midlife blood pressure is eventually the more relevant predictor of dementia development compared with hypertension in later life [28,31]. The decline in blood pressure in later life might be secondary to the brain lesions [31,35] and may give the impression of a ‘normalization’ of blood pressure values during the course of dementia progression [35], which might contribute to a lower prevalence of documented hypertension in individuals with dementia. This alternative explanatory approach is supported by our finding that the gap between dementia patients and non-demented control subjects is reduced when only community-living individuals are examined (OR = 0.90 vs. OR = 0.76) and nursing home residents – who are older and most probably at a more advanced stage of dementia – are disregarded.

Some other conditions are likely to be dementia sequelae. These include the inadequate intake of water and electrolytes [36,37], incontinence [38] and aspiration pneumonia in late-stage dementia [39,40]. Increased odds for pneumonia can also be attributed to the higher proportion of Parkinson patients among the dementia population, who are susceptible to aspiration pneumonia as well. Furthermore, neurodegenerative changes in the brain probably affect sleep regulation [41,42], as well as disorganization of the network that controls locomotion, leading to impaired gait timing and postural control [43]. This is one explanation for a difference in fall and fracture rates.

Yet, most of the comorbidity complexes inversely associated with dementia in our study have not been investigated very often. Sources of bias together with the lack of data result in contradictory findings and make it hard to explain the inverse connections. An exception might be diseases of the musculoskeletal system and connective tissue. They usually manifest in pain, and thus could be negatively associated with dementia because of altered pain processing [44]. The inappropriate communication of pain is another likely reason that may contribute to the negative association. With progressing dementia, the capabilities of expressing and especially verbalizing pain decrease step by step. Thus, individuals with dementia might face difficulties in raising awareness of their physical pain. Deficits in communication also seem to be a possible explanation for the lower prevalence of diagnosed vision and hearing impairment in individuals with dementia.

Second, the observed associations could be based on differing utilization patterns of individuals with and without dementia. Only if a patient presents to a physician will his/her diagnoses be reflected in claims data. A recent German study found that incident dementia leads to an increase in service utilization, as well as that the majority of dementia patients’ physician contacts (79%) take place in the disciplines of primary care and neuropsychiatry [45]. Other studies report that the number of different physicians contacted seems to be rather low for multimorbidity patterns including dementia as well [46], and that, in comparison with a control population, a larger proportion of dementia patients presents to a general practitioner, but a smaller proportion to specialists (neurologists and psychiatrists excluded) [47]. This applies especially to ophthalmologists, gynaecologists, otolaryngologists, cardiologists and orthopaedists, who are all specialists in disease groups that are mainly inversely associated with dementia in our study. If the fewer visits do not in fact correspond with fewer health problems in these specialty areas, the amount of comorbidity experienced by dementia patients in these groups is underestimated. General practitioners should consider more carefully when referral of a dementia patient to a specialist is necessary.

The third aspect is closely related to the second one. Documented morbidity does not always match actual morbidity. When Löppönnen et al. [12] compared recorded diseases with an additional clinical examination they found that more dementia patients than control subjects were actually underdiagnosed. This seems perfectly reasonable as demented individuals probably have greater difficulty in communicating their symptoms and, in the presence of a severe health problem such as dementia, physicians might overlook or simply ignore less severe diseases. Moreover, physicians might come to the conscious decision not to treat these comorbidity complexes based on the assumption that therapeutic interventions for these not per se life-threatening conditions reduce the dementia patients’ remaining quality of life disproportionately compared with the beneficial effects of treatment. However, some kind of therapeutic nihilism that therapy of these comorbidity complexes in conformity with guidelines is less worthwhile in a cognitively impaired population also cannot be fully excluded. The lower rate of vision and hearing problems, arrhythmias, hypertension, varicosis and musculoskeletal diseases in our study matches this hypothesis.

In this context, some comorbidity complexes stand out from our analysis. Hypertension is the most surprising example of a lower documented prevalence rate, as one would expect at least similar occurrence of hypertension in the case and control group. We have already provided possible explanations in a previous paragraph, yet those are unconfirmed hypotheses. Therefore, we also want to bring up potential under-diagnosis of hypertension. We highlight hypertension as it is a risk factor for a number of other conditions such as stroke or cardiovascular disease, and preventing these complications will certainly influence the patients’ quality of life.

Supposedly better vision and hearing in people with dementia also seems implausible. Diagnosing diseases of the eye and ear usually requires the patients’ participation, and the process is more difficult and exhausting when dealing with a dementia patient. In consideration of the extra effort, physicians might discontinue diagnosis of these seemingly less severe diseases. However, seeing and hearing abilities are important for taking part in daily life. Additionally, studies from various settings suggest that non-pharmacological, multicomponent interventions have the potential to slow down the decline in cognitive function and support the capacity to carry out Activities of Daily Living [48,49]. Not taking care of vision and hearing impairment means taking away the dementia patients’ chance of participating properly in these types of interventions, and thus taking away an opportunity to slow down the course of the disease.

### Comorbidity and gender

Basically, men with and without dementia do not have a higher comorbidity burden than females with and without dementia, but there is a gender-specific comorbidity profile. The comorbidity patterns of male and female individuals observed in our study are similar to patterns found by other authors who assessed gender-specific multimorbidity [16,46]. Women both with and without dementia suffer more frequently from osteoporosis and thyroid dysfunction as well as from mental health problems, but less frequently from cancer and cardiovascular diseases.

Yet, the effect of gender on the case–control comparison was striking. When comparing cases and control subjects for the male and female population separately, gender did have a significant influence on the ORs for half the comorbidity complexes. Also, except for four conditions (fractures/injuries, purine/pyrimidine metabolism disorders, cancer and anaemia), the ORs of the male sample exceeded the corresponding ORs of the female sample for the respective comorbidity complex. This implies that the documented prevalence of comorbidity complexes is more similar between female cases and control subjects. For example, women with dementia had an OR of 3.01 of being diagnosed with incontinence, whereas the OR of a male dementia patient of being diagnosed with incontinence was 4.19. Van den Bussche et al. [3] also found that, compared with subjects without dementia, men with dementia have a higher relative risk of multimorbidity than women with dementia (RR 4.9 vs. 3.4).

To our knowledge, other studies investigating dementia and comorbidity did not focus on gender differences. This makes our finding even harder to explain. We did not find sound literature providing explanations for comorbidity differences based on the different physiology of men and women with dementia, but dementia research is far from complete. Hence, the effect of dementia on the development of other medical conditions might indeed differ for men and women, despite corresponding evidence is lacking.

There might also be differences in the diagnosis of comorbidity in men and women. We assume that the personal interaction between male patients and their physicians differs in comparison with female patients. One factor might be that the majority of physicians are males themselves, which might ease talking about sensitive issues. Moreover, symptoms of diseases (e.g. cardiac infarction) have mainly been deduced from male patients; thus physicians might be more aware of the symptom profile in male patients than in female ones. As an additional factor, one might argue that men might communicate their interests with more impetus than women. However, as far as we are able to judge, these effects apply to men both with and without dementia and might rather explain different prevalence rates between men and women than different ORs in the gender-stratified case–control comparison.

Last, differences in health care service utilization could also be an explanation. We hypothesize that men only see the need to visit a physician when a disease becomes manifest [50], whereas females see more different physicians and report on their health problems more frequently [45]. This could make the male control subjects look healthier than they really are. Manifestation of dementia increases services utilization [45,50] and, consequently, physicians also document other, previously ignored health problems in the male population. The fact that male dementia patients probably still live with a spouse who is vigilant about regular physician’s visits, whereas older women are more often single-living and have no advocacy caring for their health care-seeking behaviour very likely adds to the effect, too.

### Comorbidity and living environment

For our analysis of comorbidity in the community setting, nursing home residents were excluded. This probably implies the exclusion of people with severe disease. As 42.4% of dementia cases but only 3.6% of control subjects were in or moved to an institution in 2006, the restriction concerns mostly advanced-stage dementia patients, who again carry particular comorbid conditions. This explains the decrease in ORs for comorbidity complexes that are common in late-stage dementia, such as pneumonia, incontinence, fluid disorders or fall risks and also for Parkinson’s disease. The ORs for most other comorbidity complexes are comparable to the ORs in the main analysis. Slight differences may be attributed to frame conditions such as differing medication use or differing amounts of care and supervision.

### Strengths and weaknesses

Using claims data from a German SHI fund for analyses is associated with some intrinsic challenges, as pointed out by Schubert et al. [51].

First, for historic reasons, the distinct SHI funds cover different clienteles. Our analyses used data from the AOK Bavaria SHI fund, which is the market leading fund in the study region. It is known that the AOK SHI fund has a comparatively high proportion of insurants with low income, low educational level and poor health status [52]. Thus, the observed prevalence rates most likely overestimate the real prevalence within the entire population aged 65 years and older. However, it has to be assumed that the effect of overestimation affects the various comparison groups in a similar way; thus the presented ORs seem to be better transferable than the prevalence rates themselves.

Another challenge is that documented diagnoses cannot be validated via clinical examinations; hence their accuracy remains uncertain. It cannot be fully excluded that physicians misdiagnosed or miscoded some comorbid conditions. To account for this uncertainty, we conducted sensitivity analyses within which the multiple documentation of comorbid conditions was required. This more strict approach towards comorbidity hardly altered the prevalence rates compared with the main analysis where a comorbid condition needed to be documented only once. Hence, as per our point of view, miscoding and misclassification seem not to be a substantial source of bias.

Claims data lack information on disease severity. Therefore, it was (1) not possible to evaluate whether the severity level of documented comorbid conditions was comparable between our study groups and (2) whether different stages of dementia were associated with specific comorbidity profiles. Additionally, differential diagnoses of dementia subtypes are not made forcefully. In consequence, different subtypes of dementia are documented insufficiently, and mostly ‘unspecified dementia’ (ICD-10 code ‘F03’) is reported. Hence, we decided against a stratified analysis for different subtypes of dementia. Therefore, probable differences in the comorbidity profile of individuals with vascular dementia and individuals with a neurodegenerative form of dementia have been ignored.

Besides these more content-related aspects, it has to be mentioned that, owing to the large sample size, even small differences in the documented prevalence of comorbidity become statistically significant even though they might not per se be clinically relevant. We tried to address this issue by reporting adjusted prevalence rates, ORs and 95% CIs in addition to p-values in order to allow a more comprehensive judgment of our findings.

In contrast to these drawbacks, claims data have some convincing advantages.

First, diagnoses can cover the entire range of existing ICD-10 codes and are not limited to a restrictive set of common codes. Therefore, our analyses were able to investigate the relevance of less common comorbidity complexes that had been neglected in previous studies, such as the entire range of musculoskeletal disorders, vision and hearing loss or fall risks.

Second, to assign individuals to comorbidity groups, hospital diagnoses as well as outpatient diagnoses were considered. Hence, we believe we have more comprehensively captured the effective patient clientele. In particular, research assessing comorbidity based on inpatient diagnoses [4,5,7,9,10] most probably underestimates true prevalence rates because hospital treatment is only sought by a minority of patients (within our sample: 27.1% of individuals without and 40.2% of individuals with dementia).

Moreover, selection bias is restrained, as claims data-based samples also include the oldest of the old, frail individuals and especially institutionalized individuals who are often not captured in primary data-based studies. As a consequence, the variance in comorbidity profiles in different residential settings can be evaluated. To our knowledge, the effect of care setting on the comorbidity profile of individuals with and without dementia has so far not been investigated in a comparably comprehensive manner as within our own study.

This also applies to the gender-stratified comparison. The body of evidence on gender-specific comorbidity profiles in individuals with dementia is small. In particular, we found no other studies that investigated a possible interaction between the documentation of a comorbid condition and gender. Our study did, and revealed a significant influence of male gender on the documentation of many comorbidity complexes. However, our study describes some possible explanatory approaches for this observed phenomenon, but it could not unambiguously provide the reasons why the comorbidity gap between male cases and control subjects is more pronounced than that between female cases and control subjects. This illustrates the need for further more qualitative research in this regard.

## Conclusion

Our data confirm known dementia risk factors and sequelae that should be managed adequately and in due time. We also point to conditions that might be prone to under-diagnosis and lack of treatment, such as hypertension, problems with vision and hearing or musculoskeletal disease. Physicians should be especially alert to these conditions, which seem to be related to complications, more rapid dementia progression and reduced quality of life. These comorbidity complexes might represent a starting point in the development of strategies to prevent comorbidity-associated negative health outcomes and to improve care for individuals with dementia. To further identify undiagnosed comorbid conditions and enhance their diagnosis and treatment, more research in dementia patients focusing on under-diagnosis as well as on their health care-seeking behaviour is needed. In this regard, special focus on gender-specific needs and attitudes towards comorbidity seems to be desirable.

## Competing interests

The authors declare that they have no competing interests.

The analysis of comorbidity complexes was conducted as an add-on to the IDA project, which was initiated and financed by the Federal Association of the AOK, the AOK Bavaria (health insurer) and the research-based pharmaceutical companies Eisai and Pfizer. For LS, RH (Helmholtz Zentrum München) and EG (Department of Medical Psychology and Medical Sociology, Friedrich-Alexander-Universität Erlangen-Nürnberg), their institutions received support from the funding organizations for the submitted work.

## Authors’ contributions

All authors were involved in the conception of the research and decided on research question and study design. KB performed the statistical analysis, interpreted the data and drafted the manuscript. LS drafted the statistical models and acted as the corresponding author. Together with RH, she conceived the study, provided statistical support and advised on health care system-related issues. EG was the main contact person for medical issues. All co-authors proofread the manuscript critically and approved its final version.

## Pre-publication history

The pre-publication history for this paper can be accessed here:

http://www.biomedcentral.com/1471-2318/14/10/prepub

## Supplementary Material

Additional file 1 Source of origin got considered comorbidity complexes. Click here for file

Additional file 2 Diagnosed comorbidity complexes of community-living dementia patients and control subjects stratified by gender. Click here for file
