# Supplementary material for: A claims data-based comparison of comorbidity in individuals with and without dementia
Source: BMC Geriatr. 2014 Jan 28;14:10. doi: 10.1186/1471-2318-14-10 (PMC3909381; doi:10.1186/1471-2318-14-10)
Supplement: Additional file 1 — Source of origin got considered comorbidity complexes. [file 1471-2318-14-10-S1.pdf]

Additional file 1: Source of origin got considered comorbidity complexes

The final 30 groups were chosen

a) because of being among the 15 most prevalent groups in the case or control group (prevalence)
b) because at least 5 out of 15 similar publications (references [5-10, 12, 16-23]of the paper) also mentioned the diagnosis group (literature) or
c) after thoroughly appraisal of their clinical relevance (discussion)

The ICD codes for constructing the disease complexes refer to references Schäfer et al. (reference 16), Löppönen et al (reference 12),Ziegler (reference 22) and Borchelt (reference 23), which have been partially amended by acute forms of the conditions.

Nr	Diagnosis Group	Source of origin	ICD 10 codes
1	Cancer (all) (C00-D48)	prevalence, literature	16
2	Anemias (D50-53, D55-D64)	literature	16+ acute forms
3	Thyroid Dysfunction (E01-07)	prevalence, literature	16+ acute forms
4	Diabetes (E10-E11)	prevalence, literature	16
5	Disorders of lipoprotein metabolism and other lipidaemias (E78)	prevalence	16
6	Fluids/electrolyte disorders (E86, E87, R60)	prevalence	23
7	Psychotic/neurotic disorders (F20-29, F40-48)	discussion	ICD 10 GM Version 2006
8	Depression (F32-33)	prevalence	16
9	Insomnia (F51, G47)	discussion	16
10	Severe vision reduction(H17-18, H25-28, H31, H33, H34.1-.2, H34.8-.9, H35-36, H40, H43, H47, H54)	prevalence	16+ acute forms
11	Severe hearing loss (H90, H91)	discussion	16+ acute forms
12	Parkinson (G20-22)	literature	16
13	Hypertension (I10-I15)	prevalence, literature	16
14	CAD (I20-25)	prevalence, literature	12, 22
15	Cardiac arrhythmias (I44-49)	prevalence, literature	16+ acute forms
16	Cardiac insufficiency (I50)	prevalence, literature	16
17	Atherosclerosis/Peripheral arterial occlusive disease (I65-66, I67.2, I70, I73.9)	prevalence, literature	16
18	Cerebral ischemia/Chronic stroke (G45, I60-64, I69)	prevalence, literature	16
19	Lower limb varicosis (I83, I87.2)	prevalence	16
20	Pneumonia (J12-18)	discussion	22

21	Asthma/COPD (J40-47)	literature	16+ acute forms
22	Arthritis (M02, M05-M06, M08, M10, M11-M13)	discussion	12
23	Joint Arthrosis (M15-19)	prevalence	16
24	Purine/pyrimidine metabolism disorders/Gout (E79, M10)	Top 15 both	16
25	Low back pain (M40-48, M50-54)	Top 15 both	16+ acute forms
26	Osteoporosis (M80-82)	Discussion	16
27	Renal insufficiency/failure (N17-19)	Literature	16+ acute forms
28	Incontinence (N39.3, R32, R15)	Top 15 case	16+ fecal incontinence
29	Fractures and Injuries (S00-T14)	Top 15 both	ICD 10 GM Version 2006
30	Fall risk and dizziness (R26, R29.6, R42, H81, H82)	Discussion	23
