# Supplementary material for: A claims data-based comparison of comorbidity in individuals with and without dementia
Source: BMC Geriatr. 2014 Jan 28;14:10. doi: 10.1186/1471-2318-14-10 (PMC3909381; doi:10.1186/1471-2318-14-10)
Supplement: Additional file 2 — Diagnosed comorbidity complexes of community-living dementia patients and control subjects stratified by gender. [file 1471-2318-14-10-S2.pdf]

**Additional file 2: Diagnosed comorbidity complexes of community-living dementia patients and control subjects stratified by gender**

| | Men (N=9,973; 1,684 cases; 8,289 control subjects) | | | Women (N=23,146; 3,840 cases; 19,306 control subjects) | | |
|---|---|---|---|---|---|---|
| Diagnosis group | Case (%) | Control (%) | OR* | Case (%) | Control (%) | OR* |
| **Neoplasms** | | | | | | |
| Cancer (all) | 37.4 | 35.9 | 1.03 (0.92-1.15) | 23.4 | 24.1 | 0.97 (0.89-1.05) |
| **Diseases of the blood and blood-forming organs** | | | | | | |
| Anaemia | 26.5 | 19.8 | 1.41 (1.25-1.60) | 24.7 | 18.8 | 1.40 (1.29-1.52) |
| **Endocrine. nutritional and metabolic diseases** | | | | | | |
| Thyroid dysfunction | 19.7 | 17.7 | 1.10 (0.97-1.26) | 30.4 | 32.6 | 0.93 (0.86-1.00) |
| Diabetes | 49.6 | 40.0 | 1.41 (1.27-1.57) | 44.7 | 38.6 | 1.29 (1.20-1.38) |
| Disorders of lipoprotein metabolism and other lipidaemias | 51.3 | 51.3 | 0.95 (0.86-1.06) | 47.4 | 52.2 | 0.86 (0.80-0.92) |
| Fluids/electrolyte disorders | 29.1 | 16.9 | 1.96 (1.74-2.21) | 29.6 | 20.3 | 1.61 (1.49-1.74) |
| **Mental and behavioural disorders** | | | | | | |
| Psychotic/neurotic disorders | 20.8 | 13.4 | 1.66 (1.45-1.89) | 27.6 | 19.8 | 1.62 (1.49-1.75) |
| Depression | 25.4 | 10.1 | 2.94 (2.58-3.35) | 34.3 | 20.0 | 2.11 (1.96-2.28) |
| Insomnia | 16.3 | 9.5 | 1.81 (1.56-2.10) | 17.2 | 13.0 | 1.36 (1.23-1.49) |
| **Diseases of the nervous system** | | | | | | |
| Parkinson’s disease | 14.8 | 2.9 | 5.73 (4.75-6.92) | 8.2 | 2.4 | 3.57 (3.08-4.14) |
| **Diseases of the eye and ear** | | | | | | |
| Severe vision reduction | 43.1 | 47.3 | 0.81 (0.73-0.90) | 41.9 | 51.3 | 0.68 (0.64-0.73) |
| Severe hearing loss | 22.7 | 20.7 | 1.09 (0.96-1.24) | 18.8 | 17.3 | 1.06 (0.97-1.16) |
| **Diseases of the circulatory system** | | | | | | |
| Hypertension | 75.4 | 73.1 | 1.03 (0.92-1.16) | 77.0 | 79.7 | 0.83 (0.76-0.90) |
| Coronary artery disease (CAD) | 48.8 | 42.9 | 1.22 (1.10-1.35) | 38.6 | 36.4 | 1.06 (0.98-1.13) |
| Cardiac arrhythmias | 35.1 | 31.6 | 1.14 (1.02-1.27) | 28.8 | 27.4 | 1.03 (0.96-1.12) |
| Cardiac insufficiency | 39.2 | 25.8 | 1.84 (1.64-2.05) | 44.4 | 33.9 | 1.45 (1.35-1.56) |
| Atherosclerosis/peripheral arterial occlusive disease | 50.9 | 32.3 | 2.09 (1.88-2.33) | 43.1 | 29.8 | 1.75 (1.63-1.87) |
| Cerebral ischaemia/chronic stroke | 38.0 | 15.6 | 3.23 (2.88-3.62) | 26.7 | 13.8 | 2.20 (2.03-2.39) |
| Lower limb varicosis | 19.2 | 16.5 | 1.17(1.02-1.34) | 29.8 | 29.5 | 1.02 (0.95-1.10) |

| | | | | | | |
|---|---|---|---|---|---|---|
| **Diseases of the respiratory system** | | | | | | |
| Pneumonia | 11.9 | 6.2 | 1.98 (1.66-2.35) | 7.4 | 4.4 | 1.69 (1.47-1.95) |
| Asthma/chronic obstructive pulmonary disease (COPD) | 35.0 | 31.0 | 1.16 (1.04-1.30) | 23.3 | 22.9 | 1.02 (0.9-1.11) |
| **Diseases of the musculoskeletal system and connective tissue** | | | | | | |
| Arthritis | 14.9 | 15.3 | 0.95 (0.82-1.10) | 12.1 | 13.1 | 0.92 (0.83-1.02) |
| Joint arthrosis | 38.3 | 39.6 | 0.91 (0.82-1.02) | 47.3 | 50.6 | 0.87 (0.81-0.93) |
| Purine/pyrimidine metabolism disorders/gout | 35.3 | 36.9 | 0.90 (0.81-1.01) | 25.0 | 24.9 | 1.00 (0.92-1.08) |
| Low back pain | 54.5 | 55.9 | 0.90 (0.81-1.00) | 58.0 | 60.8 | 0.91 (0.85-0.97) |
| Osteoporosis | 9.1 | 6.5 | 1.40 (1.16-1.69) | 31.6 | 29.3 | 1.11 (1.03-1.19) |
| **Diseases of the genitourinary system** | | | | | | |
| Renal insufficiency/failure | 29.2 | 20.2 | 1.59 (1.41-1.79) | 17.8 | 14.2 | 1.26 (1.15-1.38) |
| Incontinence | 43.0 | 17.5 | 3.46 (3.09-3.88) | 45.5 | 25.6 | 2.38 (2.22-2.56) |
| **Injuries. fractures and fall risks** | | | | | | |
| Fractures and injuries | 35.1 | 27.6 | 1.38 (1.23-1.54) | 40.2 | 32.2 | 1.39 (1.29-1.49) |
| Fall risk and dizziness | 24.9 | 15.2 | 1.80 (1.59-2.05) | 29.0 | 22.0 | 1.41 (1.30-1.52) |

*ORs are adjusted for age.
